# Institutional Notes and News

**Published:** 1910-11-26

**Authors:** 


### Institutional Notes and News.

**Bolton Infirmary**

The appeal of the committee of the Bolton Infirmary and Dispensary is noteworthy from the fact that great efforts are being made locally to enlist the favour of a King Edward Memorial on behalf of the institution, and from the nature of the appeal itself. This is carefully reasoned, and bases its request for subscriptions on the following grounds: The fact that the builders of the infirmary, now twenty-seven years old, clearly were unprepared for the future need for extension; that temporary measures, such as the increased number of beds placed in certain wards, have now been exhausted; that the increased population and the developments of modern surgery make extension and improvement imperative. But readers of The Hospital have not forgotten the Report on Bolton Infirmary which appeared on p. 574 of our issue of February 12 last, where all these points are dealt with, and where a high tribute was paid to the ability and many years' service of the Secretary, Mr. James Briscoe. A month later The Hospital was able to report that the committee of the hospital had resolved (p. 700, March 12 issue) to call in expert assistance. Dr. Mackintosh, of Glasgow, gave this, and his recommendations have been compressed into four parts in the appeal, as follows: (1) Internal structural alterations; (2) a detached building as a nurses' home; (3) a new wing at the south-west corner, of two storeys, for twenty-four beds; (4) a similar wing at the south-east corner, providing additional accommodation for children. We feel sure that the statesmanlike way in which the hospital authorities at Bolton have faced the situation, which was made clear in our Report of eight months ago, will bring them sympathy and assistance, and enable them to carry out Dr. Mackintosh's scheme. Nothing makes the public respect an institution more than the frank acknowledgment of its defects, if it is coupled with such prompt attempts as these to remedy them.

**Cardiff Infirmary.**

In The Hospital of October 22, page 119, an account was given of Colonel E. M. Bruce Vaughan's eloquent appeal on behalf of the Cardiff Infirmary. There we noted his argument that the Welsh National Memorial to the late King should include the support of "the greatest charity in Wales." To show that Colonel Bruce Vaughan is still very much alive to this important point, we are happy to quote from a speech he made the other day at a meeting of the Board of Management. He is reported to have said that it was of the greatest importance to those who felt the great responsibility of the financial position of the Cardiff Infirmary to know exactly how the money raised for the Memorial was going to be used. That thought was uppermost in the minds of many influential men in Cardiff and Glamorganshire, and they wanted to be assured, first of all, and before they contributed to the scheme, what part of the large sum of money would be devoted, say, to funds of the Welsh Medical School—so intimately associated with the Infirmary and so national in its character, and which must be the focus and centre of research and every purposeful endeavour to fight and conquer tuberculosis. Those influential gentlemen, as patriotic as any in the land, were of opinion that their first duty was at home, and without wishing to interfere in the least with the National Memorial, a fund should be raised to relieve the first institution for alleviating suffering in Wales of its oppressive burden of debt. They wished it done, first of all, in the interests of the 757 waiting patients and the 190 patients already under treatment in the hospital, and so that there should be a memorial to their late Sovereign for all time in the city of Cardiff. Colonel Bruce Vaughan knows how to drive a point home, and we feel sure that many in Cardiff will support his view of the case.

**Royal Hampshire County Hospital, Winchester.**

At the last meeting of the Governors of this hospital several alterations and additions to the statutes were passed which will have the effect of considerably shortening the present somewhat cumbersome system of the management. The most important alterations are as follows: The financial year will in future run from January 1 to December 31. The present house committee is abolished, and the Secretary will in future have larger responsibilities, dealing direct with the committee of management. The appointment of the more important officers will be in the hands of an election committee, and not in the Court of Governors as previously. In future all in- and out-patient letters of recommendation will require the signature of a medical practitioner to render them valid, and will only entitle the patient to six weeks' treatment in place of three months' as heretofore. A secretary is to be appointed who shall devote his whole time to the affairs of the hospital, and who shall have the control of all matters of management except those appertaining to the medical-surgical nursing and chaplain's departments, and for whom quarters are to be found, if possible in the building.

**The Walsall District Hospital.**

A really remarkable bazaar has been held in support of the Walsall and District Hospital, Birmingham, which produced the unexpected sum of £5,200. The recent quarterly meeting of the general committee certainly was justified in congratulating itself last week on this result. It shows two things, which we shall never grow tired of referring to, that a hospital must be mismanaging itself in some way or another nowadays if it is not the most popular institution of its district—and how popular the Walsall Hospital is can be shown best by remarking that for the bazaar only £3,000 was asked for; and, secondly, that energy can, and does every day, perform miracles. We believe it to be a safe assertion that the success of any appeal—indeed, the very sum so realised—depends precisely upon the amount of energy put into the launching of it. Walsall Hospital has paid its current debt and is now debating what to do with its unexpended balance. Mr. F. J. Cotterell, the Chairman, was supported by Mr. G. W. Warner in holding that the sum in hand was not large enough to justify the re-opening of the closed wards.

**Royal Berkshire Hospital.**

The case for the investment of legacies was put very well indeed in the recent Treasurer's report for the third quarter of the year at the Royal Berkshire Hospital, Reading. His (Colonel J. E. Broadbent, C.B.) report illustrated the above by pointing out that, though the hospital had received a very abnormal amount in legacies, £6,300, a legacy means almost invariably the death of a good subscriber, and therefore it ought to be invested, if possible, to replace in part the subscription lost. We fear, moreover, that at Reading deaths are not the only cause of the decline in the annual subscriptions to the hospital. Two years ago a building scheme was adopted at an estimated cost of £20,000; add £2,200 to this for architect's fees and equipment, and we have a total of £22,200. Towards this £11,000 has been subscribed; the other half has yet to be forthcoming. It is the County Hospital, in the county town, in the position, consequently, of being able to do the greatest work that can be done in the busiest, and, for that reason, the wealthiest centre. Cannot the authorities of the Royal Berkshire Hospital get these facts emphasised a little?

**Great Northern Hospital.**

THE "League of Roses," in connection with the Great Northern Hospital, Holloway, the idea of which originated with Mrs. D. S. Waterlow, has held its inaugural meeting. The league is to be an organisation of women, who hope to raise £1,000 a year by small sums for the support of the women's surgical ward in the Great Northern Central Hospital. Half-a-crown has to be collected or given by each member, and in this way it is hoped to gain not only the support but the sympathy of all the women of Islington and the neighbourhood for the hospital. Islington will be divided into four districts for the purposes of the league, which takes the place of and extends the scope of the half-crown league, which was started last year to assist the hospital's funds. The change of name indicates the wider aims and spirit of the new league, which is particularly fortunate in having gained the consent of her Highness Princess Victoria of Schleswig-Holstein to become its first president. Each divisional committee is asked to place out 200 collecting cards with boxes, each card to represent eight half-crowns; and thus, if they are taken up enthusiastically, each division will be able to raise £200. Next summer a festival of roses is to be held, to which all the league's friends will be asked to send roses, and at which rose badges will be distributed to the members. It is even hoped to have some morris dancing. This is a capital beginning, and it is interesting to see that just as the spirit of the King Edward Hospital Fund for London has led to the establishment of local funds in many a provincial city, so the spirit of the League of Mercy has also begun to blossom; we shall look forward to the summer, when we may expect the full opening of the Holloway rose. If any hints on morris dancing are needed we commend to Mrs. Waterlow the instructive exhibitions of this dancing now in progress at Crosby Hall.

**Odiham Cottage Hospital.**

LADY PETRE opened the other day the new Cottage Hospital at Odiham, Hampshire. A stone in the porch has the following legend: "This hospital was erected with funds generously left for that purpose by John McIntyre, M.D., of this town, and completed June 1909." The architect was Mr. A. H. Guyer, of Farnham. The following account of the origin and history of the hospital we take from a capital speech made by Mr. G. L. Brooks, who is chairman and hon. secretary of the preliminary committee. "By the will of the late Dr. McIntyre £1,000 was given to Odiham to build a cottage hospital. Unfortunately this was not free from legacy duty, which with costs reduced it to £893 14s. This sum was added to by Miss McIntyre's contribution of £500. The hospital has been built on the almshouse ground, by permission of the trustees, so that we have had to pay nothing for the site. The cost of the hospital, including the architect's fees, has been £1,273, which left in hand a balance of £219, of which £215 has been invested, this forming the total endowment at present. From the four years' old Hospital Sunday Fund had been taken the necessary money to furnish the hospital. The hospital will be governed under a scheme, drawn up by the Charity Commissioners, under which it is shown to be for the poor of Odiham and of the neighbourhood. No person suffering from infectious or incurable disease will be admitted, and private patients will be accepted for treatment, if room permitted, for not less than two guineas a week. I do not think," Mr. Brooks concluded, "that the Committee has any idea at present of what it will cost to run the hospital, but it can hardly be under £200 a year. The staff at present consists of Matron Tees and Nurse Stroud, and they have already had three patients in the hospital." We hope the matron or the secretary, when appointed, will keep us informed of the hospital's doings from time to time.

**Newark Hospital, Notts.**

THE earnest appeal for the more worthy and more general support of the Newark Hospital, Trentside, Notts, put forward by the Duke of Portland the other day, should certainly prove successful. His Grace's own example in having given recently an operation theatre proves his own belief in his appeal, and we cannot doubt that the locality will follow enthusiastically the lead which he, as the hospital's President, has given by deed and word. In his recent speech he summed up the case as follows: "The hospital had a debt at the beginning of this year which amounted to £610, and, whilst its income amounts to about £1,600, the expenditure amounts to about £1,800, thus showing an annual deficit of about £200. Perhaps some people might be inclined to say that those responsible for its management should cut their coat according to their cloth; but it would be a great pity to do so, for the Board of Management, after most full inquiry, are satisfied that they are unable to reduce the expenditure without reducing the benefits the hospital affords. Its work, too, has recently vastly increased, for 2,298 patients were treated last year, compared with 1,578 ten years ago. The Board of Management, in their responsible position, however, fully recognise that they cannot indefinitely go on incurring an annual loss of £200, and, ladies and gentlemen, the object of this meeting is to try to obtain additional annual subscriptions to that amount, and I appeal to you one and all to make this object a success. The amount received last year from annual subscriptions came to £488, given by 210 subscribers, 137 of whom live in Newark and 63 in the country districts, but at the present time I am sorry to have to say—for it does not appear very creditable to relate—that there are no annual subscribers for several of the villages which send patients to the hospital. In the first place, not only must we appeal for, but also find a much larger number of annual subscribers from both this town and the surrounding country districts. In the second place, it is desirable to try to obtain larger subscriptions from those firms which each year send a good number of patients to the hospital. In conclusion, may I remind you that I believe there is a time-honoured custom in this town which is known as 'Ringing for Gofer'? I am told that this is the time of the year the ceremony takes place, and that it dates from the reign of Queen Elizabeth, when one Gofer, a rich man, lost his way in this neighbourhood, and was saved from robbery, and perhaps murder, by hearing the bells of this town. In his gratitude he left a sum of money to pay for an annual chime of the bells. May I suggest to you that at the present time your bells should not only ring in memory of Gofer, but that their ringing should remind those who live within their sound not only of the worthy Gofer, but also of the very pressing needs of the philanthropic institution which we have met to-day to support?" Mr. W. S. Davy said that there was not the slightest justification for any adverse criticism of the hospital.

